# Pressure Measurement-Based Method for Battery-Free Food Monitoring Powered by NFC Energy Harvesting

**DOI:** 10.1038/s41598-019-53775-1

**Published:** 2019-11-26

**Authors:** Thanh-Binh Nguyen, Viet-Thang Tran, Wan-Young Chung

**Affiliations:** 1 Department of Electronic Engineering, Pukyong National University, Busan, 48513 South Korea; 2 Department of Mechanical-Electrical-Electronic Engineering, Ho Chi Minh City University of Technology, Ho Chi Minh, Vietnam

**Keywords:** Electrical and electronic engineering, Electronics, photonics and device physics, Electronic and spintronic devices

## Abstract

A novel approach for battery-free food freshness monitoring is proposed and demonstrated in this study. The aim is to track the freshness of different sorts of food such as pork, chicken, and fish during storage. To eliminate the drawbacks of conventional food monitoring methods, which are normally based on measuring gas concentration emitted from food in a container, this approach measures the gradual increase in air pressure caused by the gas emission during storage. Additionally, we aim to design a smart sensor tag that can operate in fully passive mode without an external power source. To achieve this goal, near-field communication (NFC)-based energy harvesting is utilized in this work to achieve a self-powered operation of the sensor tag. To demonstrate the feasibility of the proposed method, experiments with the above-mentioned food were tested at room and refrigerated temperatures in 2 and 4 days, respectively. For each experiment, 200 g of the target food was placed in a 2-L container with the smart sensor tag. The experiments were conducted with both rigid and flexible containers to consider real food packaging environments. The air pressure inside the container was monitored as an indicator of food freshness by a sensitive pressure sensor on the smart sensor tag. The experimental results showed a remarkable increase in air pressure, which was able to be detected with high accuracy by the pressure sensor. The fabricated battery-free smart sensor tag is small (2.5 cm × 2.5 cm) and is capable of less than 1 mW of power consumption, which is ultra-low relative to other ordinary approaches that have a power consumption that normally surpasses 10 mW. The pressure value was used to classify food freshness into different levels on a mobile display to provide food freshness status using an NFC-enabled smartphone.

## Introduction

Near-field communication (NFC) is a technology that enables simple two-way transactions between electronic devices over a short distance. Although it has existed for more than a decade^1^, NFC has just recently come into wide use with the dramatic growth of the Internet of Things (IoT). NFC consists of a reader and a tag. Once the tag is placed in the magnetic field of the reader’s antenna coil, the tag antenna harvests energy to wake the tag up, and data is then sent back to the reader through the NFC data exchange format (NDEF) message. Currently, NFC applications are widespread in contactless payment, transport cards, door access, and many other applications where simple data such as an identification number or text is exchanged promptly and safely between two devices without pairing requirements. Recently, NFC-based energy harvesting has been attracting more research attention owing to its promising potential. Specifically, the magnetic field from NFC is not only used for data communication, but also for powering up an embedded circuit with a sensor module to measure ambient environmental parameters such as temperature^2,3^, soil moisture^4^, and pH^5^. NFC-based energy harvesting provides a new and fast way to obtain data using any NFC-enabled smartphone instead of the dedicated reader normally seen in other battery-less systems.

Undoubtedly, food quality has become an enormous concern in our life due to its intimate relationship with human health^6^. Indeed, foodborne illnesses are involved in approximately 76 million cases of illness, 325,000 hospitalizations, and 5000 deaths in the United States each year. Worldwide, foodborne illness is the culprit of 600 million cases of falling ill, 420,000 deaths, and 125,000 deaths in children under five years of age every year^7^. Various symptoms are related to eating spoiled food, such as diarrhea, nausea, abdominal cramps, headache, and dizziness^8^. It is widely agreed that among many characteristics of food, freshness is a crucial factor of food quality. During storage, food freshness declines as a function of time and leads to changes in the features of meat like color, firmness, tenderness, and flavor, which affect the taste of food^9–11^. Hence, reliable monitoring systems are essential for the food industry to track food decomposition during storage, as well as during delivery or food processing to avoid detrimental issues caused by spoiled food.

Although various systems for food freshness monitoring have become available that are primarily based on embedded circuits (called electronic noses) to estimate food transformation, most of these systems are bulky, and are powered by cables or batteries^12^. This is an impediment to deployment because of the limited flexibility, and especially because chemical substances in batteries are usually perilous if ingested.

Therefore, battery-free food monitoring is a great alternative solution in this field. A number of papers have been published that proposed a system for harvesting energy from the ambient environment (such as from RF waves) for self-powered operation^13–15^. A major drawback of these works is that they focused on measuring gas concentration for monitoring freshness, which requires very high power consumption by the gas sensors (normally above 10 mW), whereas the energy harvested from RF waves is at the level of microwatts^16–25^. As a result of this problem, the system can only be activated for a very short time between long charging processes. Moreover, the gas sensors also need a long time to warm up to yield an accurate result. A question has arisen whether it is reliable to wake the sensors up and read data immediately, when the result might not be accurate or steady enough. Additionally, a dedicated reader was required in their study^13–15^ to provide power and read the measured data, which would increase the complexity and cost of the system.

In this study, we aim to develop a battery-free system that is able to evaluate food freshness with high reliability. Toward this end, a novel method is proposed for freshness monitoring that is based on the air pressure increment caused by gas emissions from rotting meat. Owing to the high resolution of the sensor and the physical sensing property, air pressure can be measured accurately with only microwatts of power used for the sensor operation. Additionally, a sensor tag is designed to harvest energy from and transfer data to a smartphone through NFC. This reduces the system cost by removing the demand of a dedicated reader as seen in other works. Furthermore, the designed sensor tag has the smallest dimensions in this field for flexible application in various scenarios, because the multi-stage rectifier and power management circuit used in other studies are eliminated. This work focuses on perishable foods such as pork, chicken, and fish, which are widely consumed to test the system usability and freshness classification. Experiments with kimchi, a Korean salted and fermented food, are also conducted to monitor the pressure increase in the kimchi package, which can lead to volume expansion and liquid leakage. Experiments with other kinds of food are beyond the scope of this study.

## Methods

### Food spoilage and conventional work

**Figure 1:**
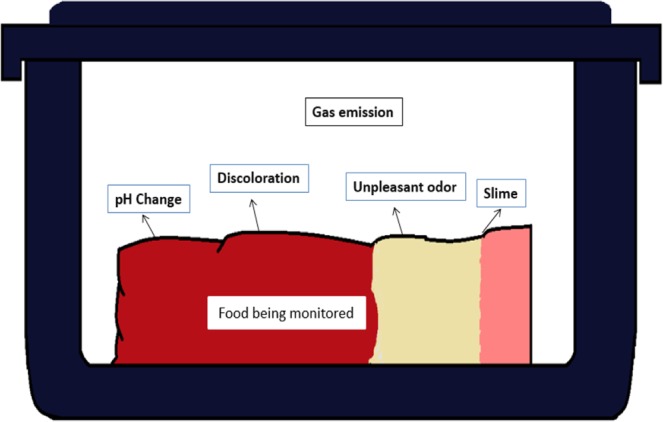
Phenomena that occur inside the food container during storage.

Food spoilage is a complex process in which both biological and chemical activities may impact and make a product unacceptable to be consumed^26^. After slaughter, food freshness declines as a function of time, pre-slaughter, post-slaughter, and storage conditions. There are many culprits responsible for this deterioration, such as attacks from enzymes, oxidation, and microorganisms. Due to these factors, various phenomena occur inside the food container (such as meat discoloration, off-odor, slime, pH level change, and gas emission, as illustrated in Fig. 1) that might be used as indicators for food freshness.

### Proposed method for battery-free food monitoring

To achieve high accuracy and low power consumption of the system simultaneously, this study employs the air pressure rise caused by gases emitted from the decaying meat inside the food container to monitor food freshness. The air pressure can be calculated by the general gas equation^27^.

1 $$PV=nRT,$$

where *P*, *V*, and *T* are the air pressure, the volume of the container, and the absolute temperature, respectively. *n* is the number of moles of gases, and R is the ideal gas constant. According to the equation, the pressure in the container depends on the number of moles of gases and the temperature inside the box. Because the storage temperature varies negligibly, it can be said that the pressure inside the container is linearly proportional to the gas concentration. Unlike most gas sensors, pressure sensors need only microwatts of power for their operation. Moreover, their fast response and compact dimensions are additional advantages in comparison with gas sensors. Therefore, evaluating food freshness based on air pressure variation would be a great alternative approach, while the drawbacks of the gas-concentration-based method mentioned above are overcome.

### Technique for self-powered operation

**Figure 2:**
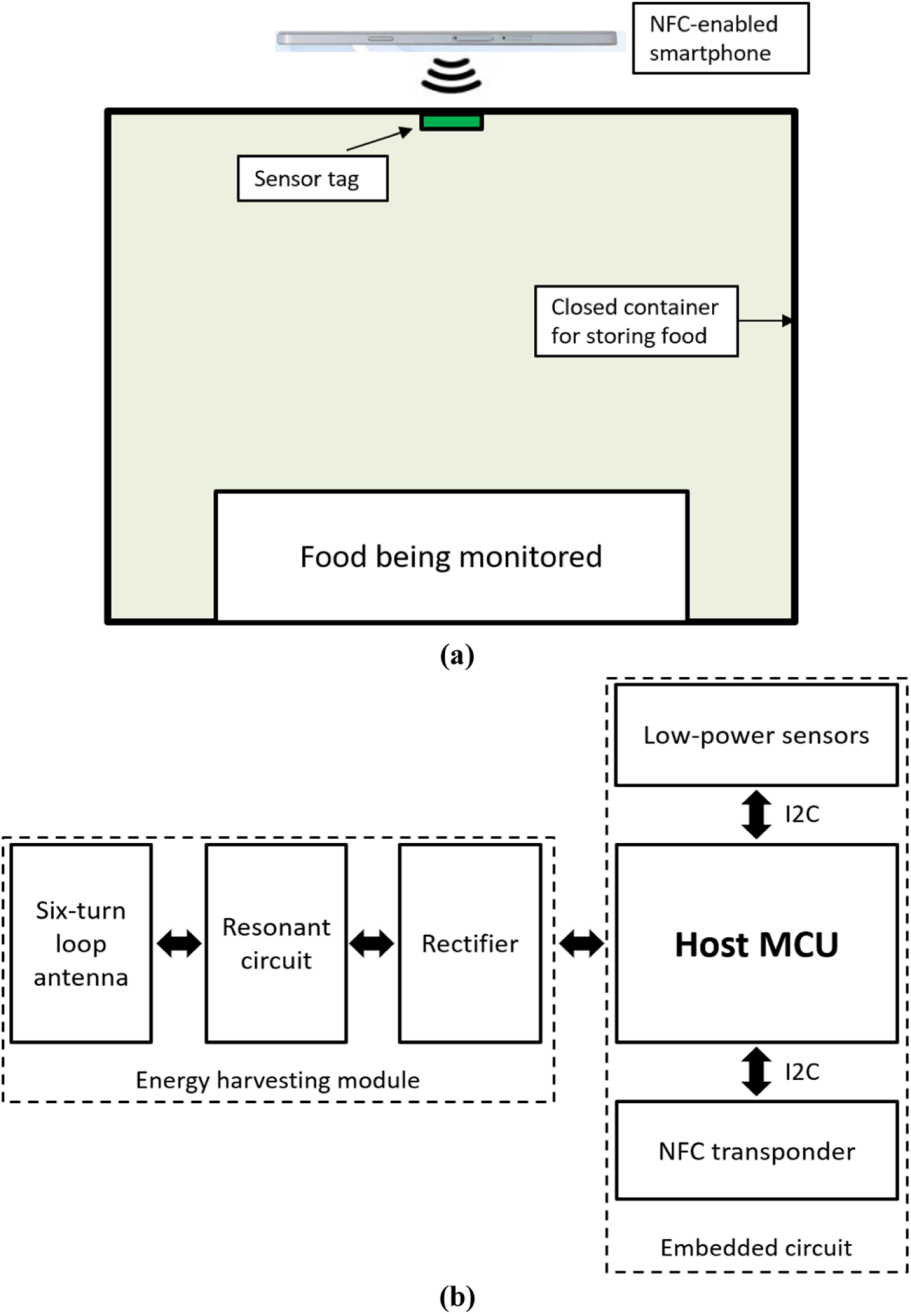
(**a**) Block diagram of the proposed system, and (**b**) block diagram of the designed sensor tag.

Figure 2 shows the block diagram of the proposed system, which consists of a container integrated with a smart sensor tag and an NFC-enabled smartphone operating as a reader. Although the harvested power from NFC varies across smartphone models, it is approximately several milliwatts to ensure that the system can work with any smartphone.

**Figure 3:**
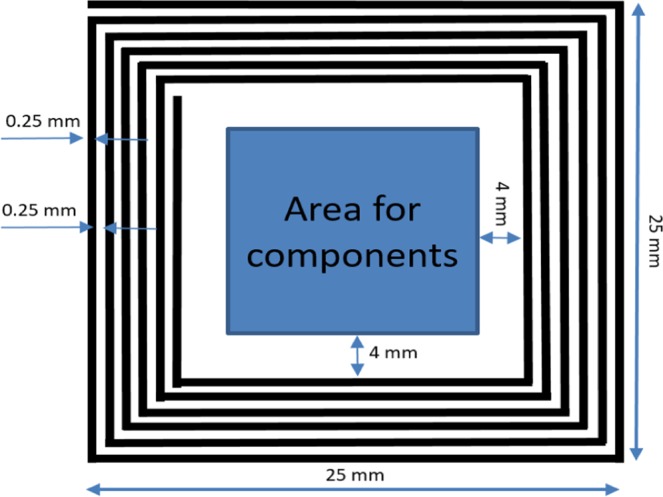
The square loop antenna design for energy harvesting and data communication.

To obtain energy from and transmit data to a smartphone via NFC, we designed a loop antenna, which is a six-turn spiral loop printed on an FR-4 substrate. The antenna dimensions are depicted in Fig. 3, with a track width and spacing of 0.25 mm. It is strongly recommended to maintain a distance of at least 4 mm between the antenna and the ground plane of the PCB to achieve the best performance of the antenna. A simulation tool (Momentum Electromagnetic Simulator, Keysight Technologies, Inc, USA) is used to design the antenna with a simulated inductance of 1.82 µH. Therefore, a tuning capacitance of 75.69 pF is needed to make the antenna resonate at 13.56 MHz. The inductance of the fabricated antenna is then measured by a network analyzer in order to find the exact value of the tuning capacitor. Furthermore, a rectifier is also designed with Schottky diodes and a 100 µF capacitor for AC to DC conversion.

A dynamic NFC interface transponder (RF430CL330H, Texas Instruments Inc., USA) is utilized in this work for data communication. It is an NFC Type 4 tag device^28^ that provides both wireless and wired interface for the connection with other devices through NFC, serial peripheral interface (SPI) or inter-integrated circuit (I2C). The SPI and I2C interfaces are also used to read or write data to the NFC data exchange format (NDEF) message in the SRAM, which can be accessed wirelessly through an ISO14443B-compliant RF interface in the device.

A low-power microcontroller (MSP430G2553, Texas Instruments Inc, USA), which has a powerful 16-bit RISC CPU, 16-bit registers^29^, is chosen as a host MCU to be integrated into the sensor tag to control the NFC transponder and the sensor module. The MSP430 family is an ultra-low-power MCU from Texas Instruments that was designed specifically for low-power applications, such as wearable or battery-less devices. This chip operates in a voltage range from 1.8 to 3.6 V, and can be configured at different clock speeds. Due to the low power consumption of the system mentioned previously, the MCU is set to operate at 1 MHz, corresponding to a current consumption of 330 µA at 3.3 V in active mode.

To measure temperature, humidity, and air pressure inside the food container, a low-power pressure sensor (BME280, Robert Bosch GmbH, Germany) is used. It is an environmental sensor, which was designed for applications where size and low power consumption are crucial constraints. The sensor is able to measure temperature, humidity, and pressure with high accuracy, and high linearity^30^. The sensor is chosen because of its compact dimension, which is integrated into an 8-pin metal-lidded 2.5 × 2.5 × 0.93 mm³ land grid array package, and because of its low current consumption (3.6 μA, at 1 Hz) that satisfy our application requirements. An additional advantage is that the sensor response time is extremely fast especially in the pressure measurement at very low noise. The BME280 sensor also supports various operating modes, which provide users with the ability to use the device in different power consumption, resolution, and performance that fits the desired application.

## Results

### Sensor tag operation analysis

**Figure 4:**
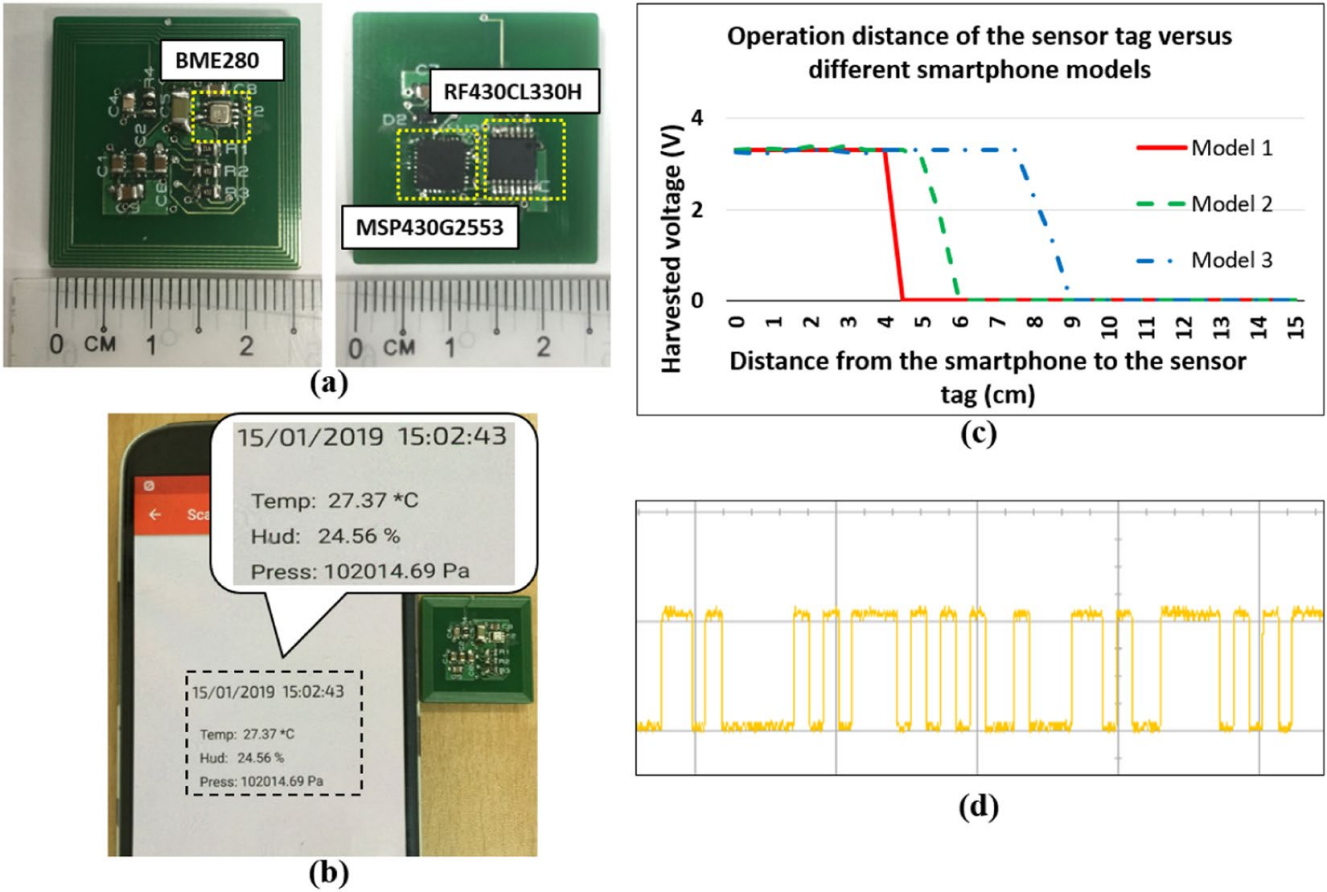
Experiments with the sensor tag. (**a**) Top and bottom layers of the PCB, (**b**) data for temperature, humidity, and air pressure read from the tag by an arbitrary smartphone, (**c**) maximum operation distance measurement of the sensor tag with different smartphones, and (**d**) data waveform at the SDA pin of the I2C communication when the tag is operating.

After fabricating the sensor tag, a set of experiments were conducted to test the operation of the designed sensor tag in fully passive mode. First, we used an arbitrary Android smartphone to read data for temperature, humidity, and air pressure from the sensor tag. Subsequently, the sensor tag was powered up using different smartphones to measure the maximum operation distance for each model to prove the system applicability. The results depicted in Fig. 4 demonstrate that the sensor tag is able to operate with all three smartphones at distances between 0 and 9 cm, depending on the NFC function of the smartphones. While it is operating, the data waveform at the I^2^C data pin is captured.

### Experiments with food

**Figure 5:**
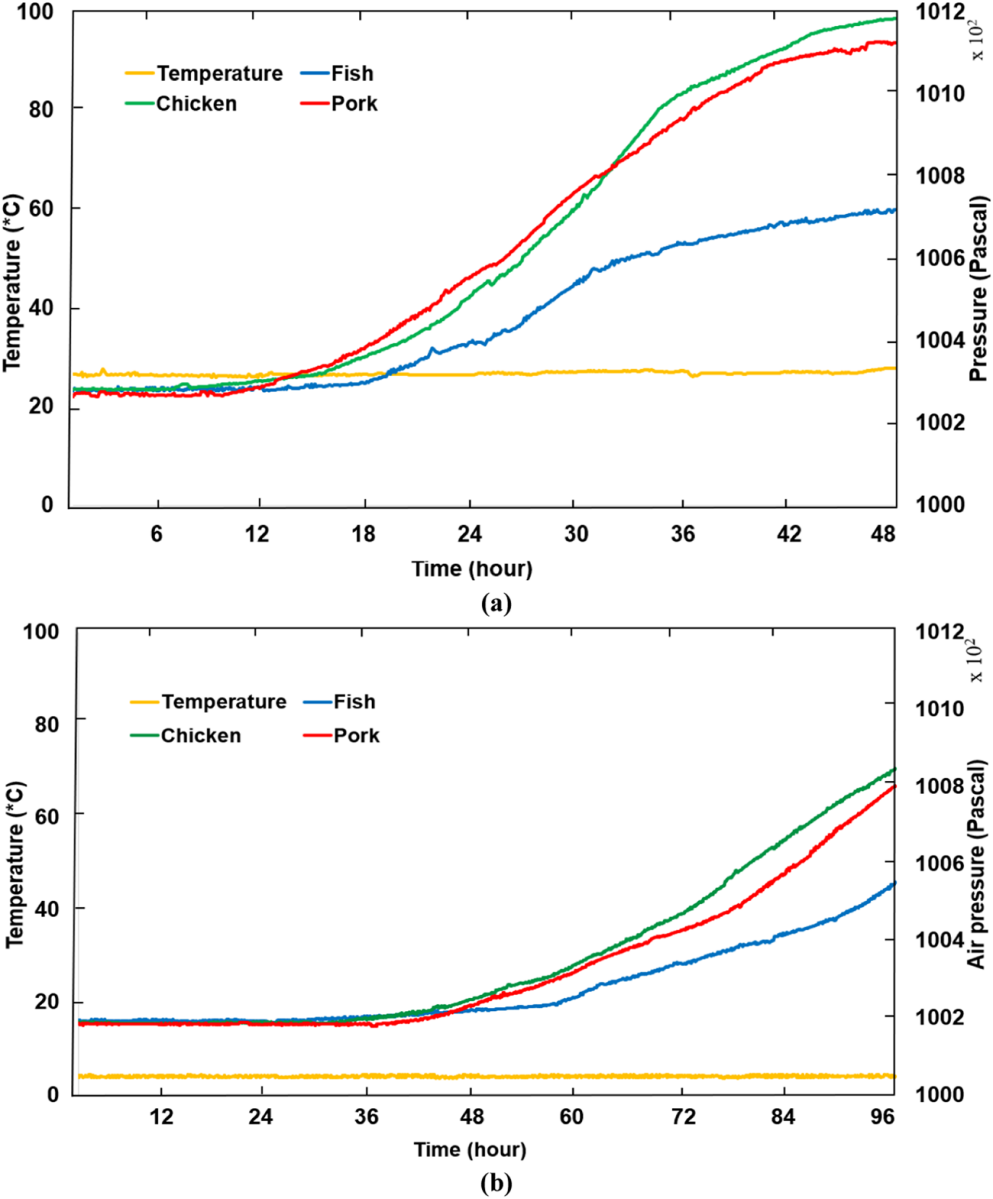
Data of package pressure increase in the experiments with pork, chicken, and fish stored in a rigid container at two storage environments. (**a**) At room temperature, and (**b**) at refrigerated temperature.

**Figure 6:**
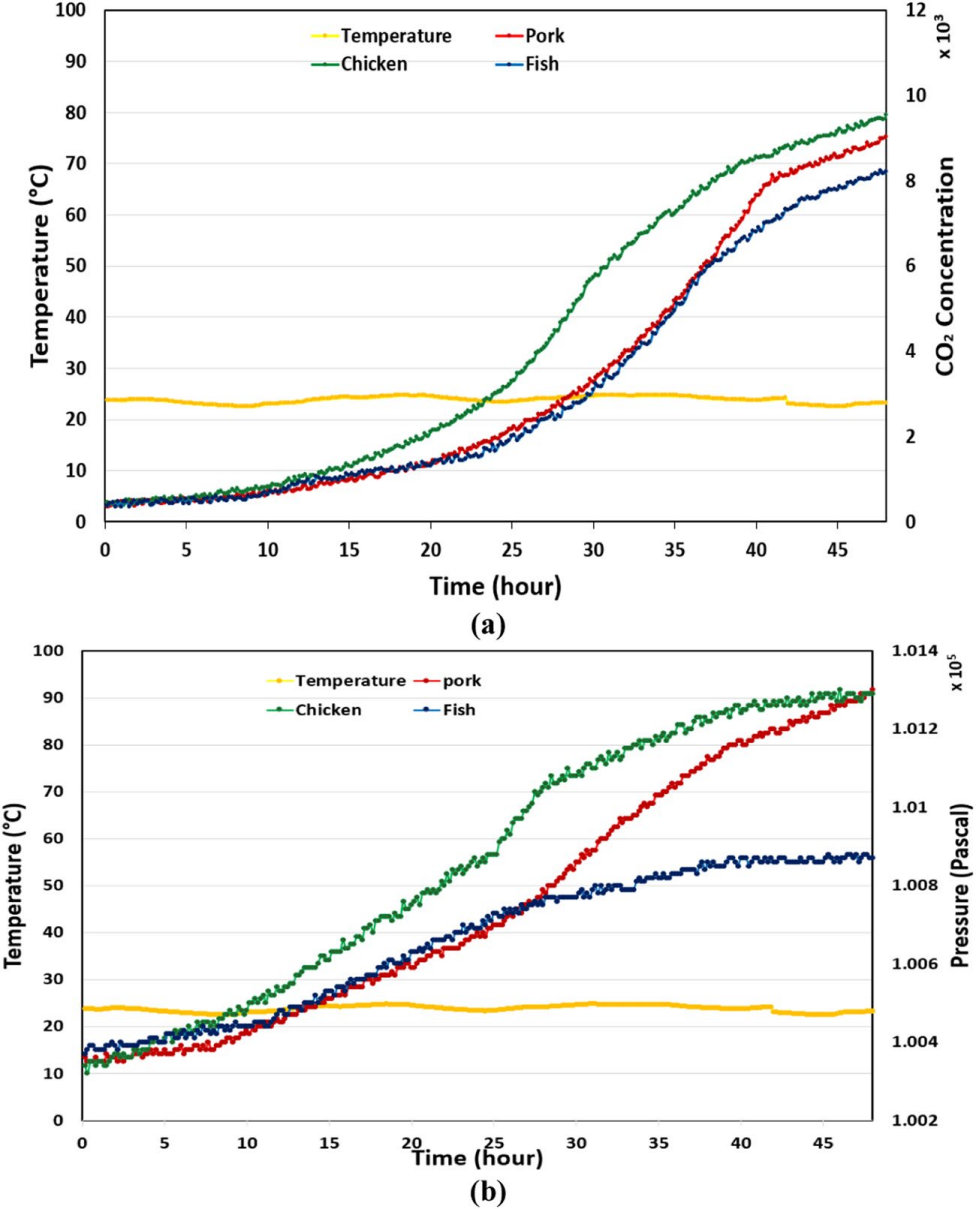
Data of package headspace pressure and CO_2_ concentration the experiments with pork, chicken, and fish stored in a plastic-flexible container at room temperature. (**a**) Headspace pressure, (**b**) CO_2_ concentration.

**Figure 7:**
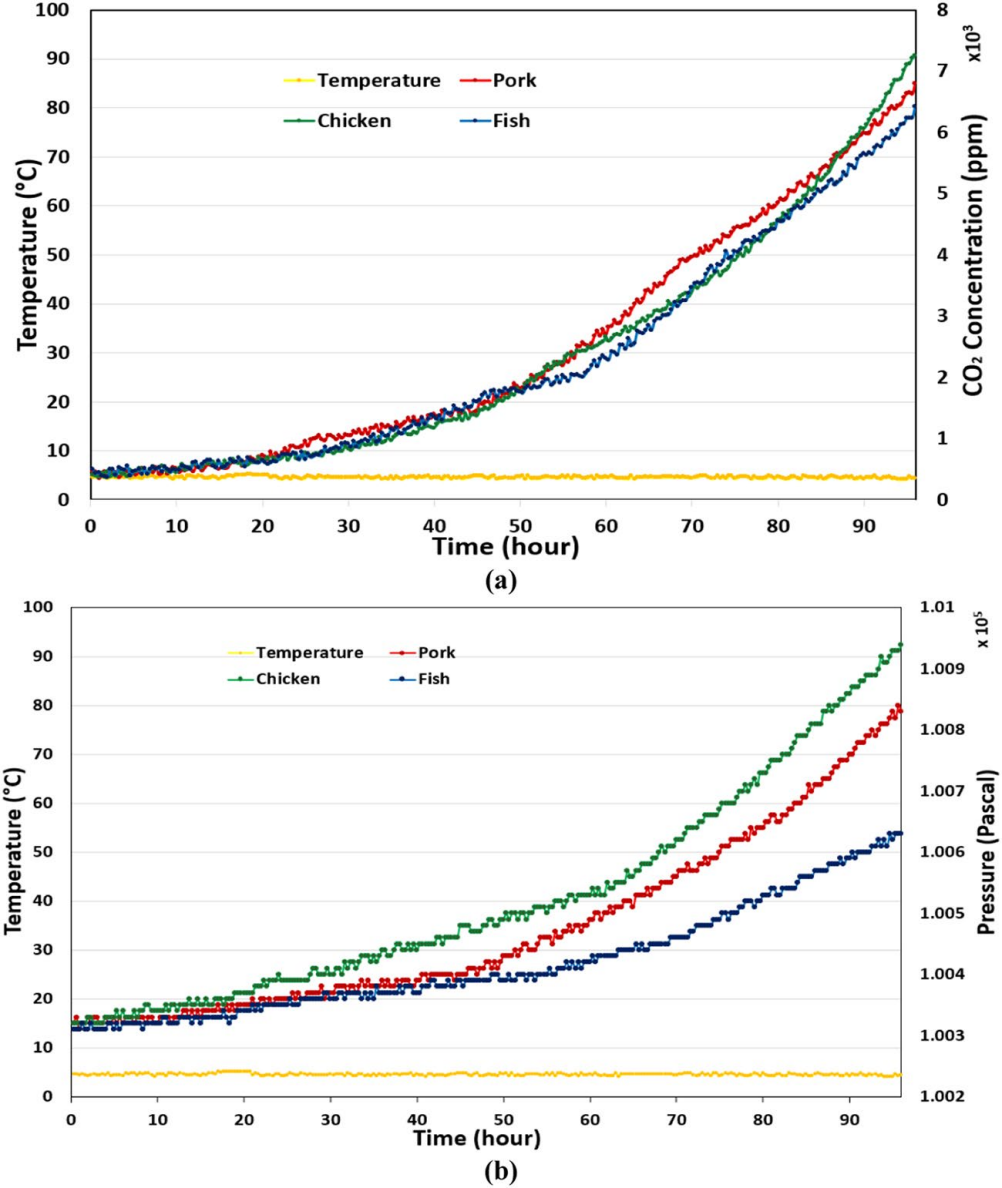
Data of package headspace pressure and CO_2_ concentration the experiments with pork, chicken, and fish stored in a plastic-flexible container at refrigerated temperature. (**a**) Headspace pressure, (**b**) CO_2_ concentration.

To demonstrate the feasibility of the proposed method, experiments with pork, chicken, and fish are sequentially carried out. For each experiment, 200 g of food is stored in a 2-L container integrated with the designed sensor tag. In this test, the sensor tag is powered by an external power source to read data every five minutes. Measured data is then passed to a computer interface via a Bluetooth module. Data for temperature and air pressure are collected in an air-conditioned room, and a refrigerator within 2 and 4 days, respectively. Figure 5 depicts the temperature and air pressure variation inside the food container in two storage environments. In the first test, it is evident that the temperature is almost steady at approximately 26 °C. In terms of air pressure, there is no change during the first 10 h, after which it starts to increase. It is clear that the air pressure increase caused by the rotting chicken and pork in this experiment is 902 and 862 Pa, respectively, which is more significant than the 440 Pa of pressure increase in the experiment with fish. The air pressure increases more slowly when food is placed in a refrigerator. After 4 d, 673 Pa and 610 Pa of air pressure increase caused by the chicken and pork are recorded, while the pressure in the experiment with fish increases by 368 Pa. The experiments were then repeated with a flexible container, which is usually used in the food industry to test the applicability of the system. In this experiment, we also store 200 g of food in a 2-L plastic flexible package in 2 and 4 days, respectively. Furthermore, we attached another sensor module to measure CO_2_ concentration, which is the main kind of gas produced from the glycolysis process of rotting meat. The experimental data are depicted in Figs. 6 and 7, which are different but have similar variation trend to the data of experiments with a rigid container.

It is important to note that the pressure resolution of the BME280 sensor is 0.16 Pa, meaning that the pressure values could be measured accurately owing to the high sensitivity of the sensor. In other words, it can be said that the proposed method, which employs air pressure variation inside the food container as a food freshness indicator, is feasible.

### Food freshness classification

**Figure 8:**
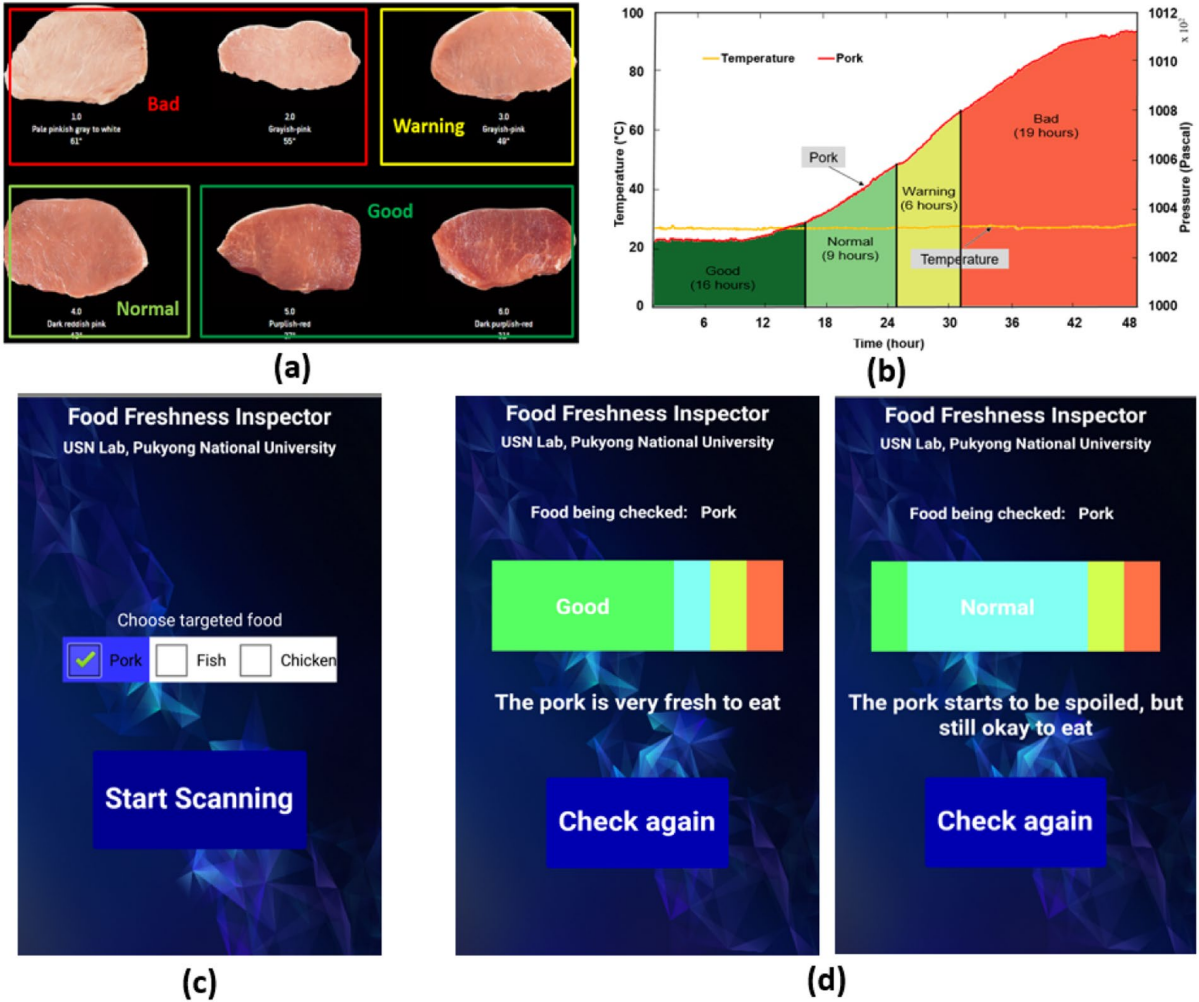
Food freshness classification result. (**a**) The six-color scale for pork, (**b**) the illustrated result of the experiment with pork, (**c**) the developed app user interface, and (**d**) the classification result displayed on the mobile phone.

Experimental results have been revealed the feasibility of using gas concentration and pressure for food freshness monitoring. In this paper, we aim to go further to classify food into different levels of freshness based on its color. Pork is used to investigate the usability of this classification. A six-color scale for pork that reflect its freshness was used in this case^31^. Photos of the standards are depicted in Fig. 8. As described in the photo, pork with dark purplish-red or purplish red color is very nutritious to eat. When the spoilage process starts, pork color turns to a dark reddish pink. At this stage, the pork is still good enough to eat. When the pork is in grayish pink, it seems to be rotten significantly. This is a warning to consumers to consider carefully before eating or buying. If the pork appears slightly grayish pink or pale pinkish gray, meaning that it is in bad condition. Throughout the storage process, the color of the pork and the pressure values inside the container are simultaneously monitored. During this process, the color is compared with the color standards to find the values of inside pressure at which the pork freshness turns from one level to another. The pressure values at those points are called thresholds. According to the result, the pork was good during the first 16 h, then it was classified as normal within the next 9 h. During the next 6 h, the pork freshness warning was seen, and subsequently the pork turned bad. The thresholds are then used in the developed mobile application for food categorization. When the food container was touched with an NFC-enabled smartphone, the app compares the received pressure with the thresholds to estimate pork freshness. The classification result is shown in Fig. 8(a).

### Experiments with kimchi

Kimchi is a traditional Korean salted and fermented food. It is currently exported to many countries in Asia, Europe, and America. During the long distribution process by cargo ship, kimchi produces a lot of carbon dioxide in the package headspace. The volume expansion due to the CO_2_ production may lead to liquid leakage, which affects the kimchi quality and package integrity.

**Figure 9:**
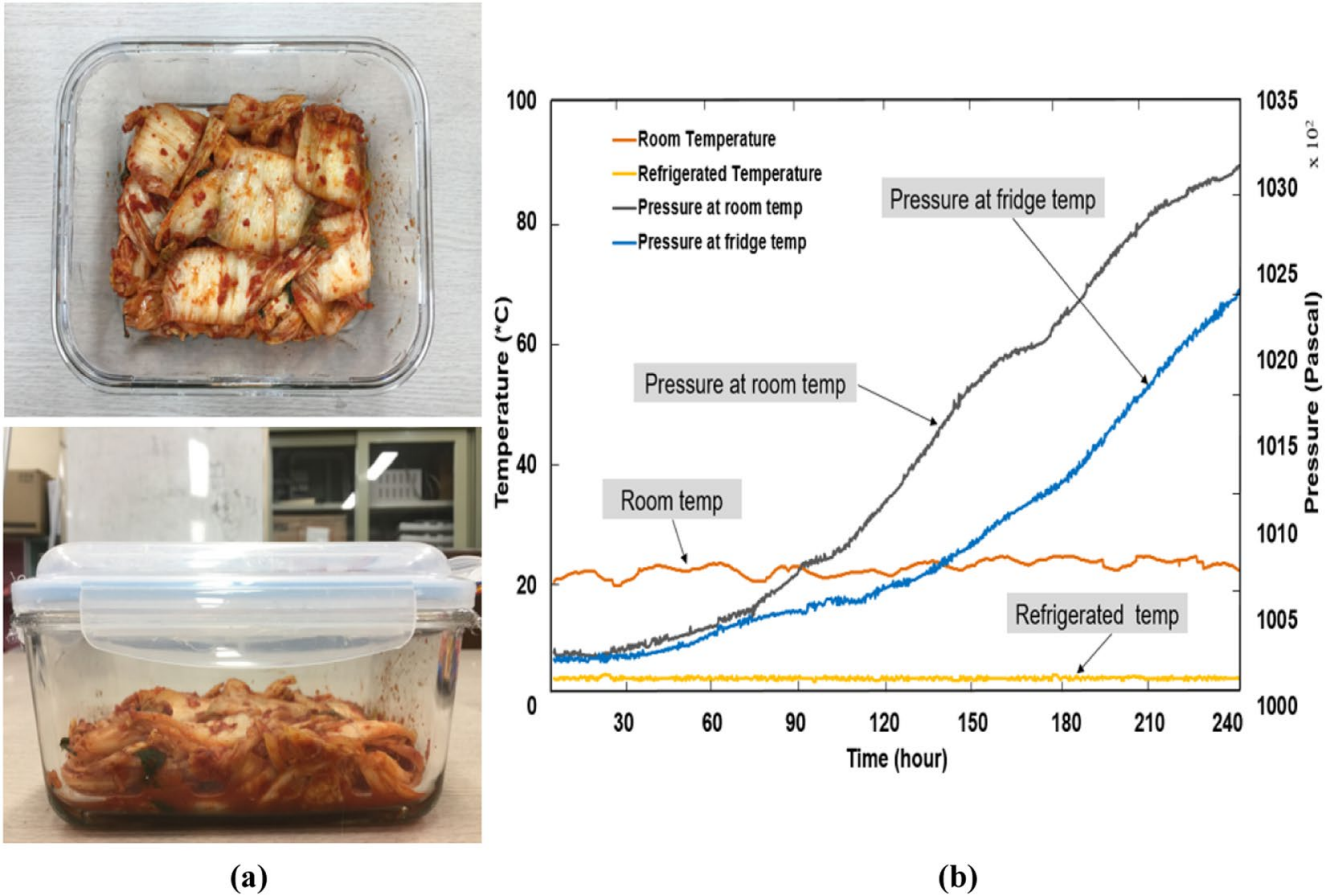
Experimental result with kimchi at room and refrigerated temperatures. **(a)** Kimchi stored in a closed container, **(b)** data of temperature and air pressure increase. Noted that temp stands for temperature.

In this study, we conducted experiments with 500 g of kimchi stored at room and refrigerated temperatures over 10 days (Fig. 9(a)). As a result, the air pressure in both experiments increased gradually with the pressure variation after 10 d is 2900 and 2000 Pa at room and refrigerated temperatures, respectively (Fig. 9(b)). The pressure variations in this experiment was much more significant than those in the previous experiments, demonstrating the feasibility of employing pressure measurement as an indicator of kimchi package over volume expansion.

## Conclusions

This paper has presented a novel method suitable for a compact, reliable, battery-free food monitoring system that are capable of estimating the level of food spoilage during storage. The proposed method, which is based on air pressure increase inside the food container, was investigated by deploying a set of experiments in two different storage environments. The experimental results proved its feasibility, because a significant increase in air pressure in each experiment was observed. The proposed method improves the system reliability, and power consumption was reduced to less than 1 mW. In addition, NFC-based energy harvesting was utilized to reduce the system cost and complexity by removing the need for a dedicated reader. With this novel approach, a sensor tag with dimensions of only 2.5 cm × 2.5 cm was designed and fabricated to read temperature and air pressure data for food freshness estimation. Air pressure values were also used to classify food freshness into different groups, and was displayed to users with the developed application. In conclusion, this study paves a new way to deploy battery-free food monitoring systems by assessing food freshness with an NFC-enabled smartphone. For future works, further experiments need to be conducted to make the system work with other sorts of food.
